# Reply to Comment on Re-Visiting Immunogenicity Associated with Botulinum Toxin Treatment. *Toxins* 2019, *11*, 491

**DOI:** 10.3390/toxins12020072

**Published:** 2020-01-23

**Authors:** Steven Bellows, Joseph Jankovic

**Affiliations:** Parkinson’s Disease Center and Movement Disorders Clinic, Department of Neurology, Baylor College of Medicine, Houston, TX 77030, USA; stbellow@bcm.edu

We appreciate the commentary on our article by Foster and Beard, both employees of Ipsen. We would like to take the opportunity to address several points raised in their commentary.

First, we agree that the relationship between secondary non-responsiveness (SNR) and neutralizing antibodies (NAbs) remains unclear. Indeed, it is the first item raised in our discussion. Detection methods for NAbs vary between different studies, complicating the interpretation of published data. We also noted that NAb-associated SNR is a rare occurrence, and SNR is “more frequently due to an insufficient dose, inappropriate muscle selection, or improper injection technique or targeting” [1]. We agree that NAb formation occurs only in “a small percentage of patients”, but a recently published study concluded that the “risk of NAb formation is still underestimated” [2]. In that study of 212 patients with cervical dystonia and known antibody status, 39 (18.4%) had antibodies detected by ELISA technique, 31 of whom also had antibodies detected by a mouse hemidiaphragm “confirmation” test. The ELISA-positive patients benefited less from BoNT than those who were antibody-negative, thus suggesting a relationship between the presence of NAb (immunoresistance) and poor clinical outcome. Although Foster and Beard state that “NAb formation rates do not appear to vary between products” there are several studies that provide contrary evidence. For example, rimabotulinumtoxinB was reported to have a 33–44% frequency of de novo NAb formation, although many patients who withdrew from the studies due to lack of efficacy did not have NAbs (detected by mouse protection assay) [3]. 

Since loss of response to BoNT is quite uncommon, the issue of immunogenicity is not frequently considered in clinical practice. There has been renewed interest in this topic, however, as a result of a paper by Albrecht et al. [4] that reported an unusually high prevalence of NAbs. In our response to the report [5] and in our review [1] we drew attention to some of the problems with the data presented in their study, including the lack of correlation between the presence of NAbs and clinical response. This poor correlation is noted in many other studies cited in our review [1,6,7]. Nevertheless, some studies provide evidence of a direct relationship between the presence of NAbs and SNR. For example, Fabbri et al. [8] reported 3.5% prevalence of NAbs among clinically responsive patients, but 53.5% among patients with SNR. 

In their commentary, Foster and Beard state that “the predominant view drawn from a number of recent systematic reviews across large numbers of studies, is that there is no significant difference in the immunogenicity rates observed with the three main type A BoNT products (abobotulinumtoxin A, incobotulinumtoxin A and onabotulinumtoxin A)”. In support, they cite reviews by Lacroix-Desmazes et al. [6], Hefter et al. [9], and Mathevon et al. [10]. However, these reviews have many limitations and warrant closer examination. The study by Hefter et al. [9] evaluated 221 patients who had received at least 10 injections for treatment of cervical dystonia. Although all were apparently still clinically responsive, 16.4% treated with abobotulinumtoxinA monotherapy had antibodies compared to 11.1% treated with onabotulinumtoxinA. The review by Mathevon et al. [10] examined NAb prevalence in patients treated with BoNT for spasticity. This indication, as discussed in our review [1], appears to have a very low rate of NAbs formation and, therefore, is not suitable for addressing immunogenicity. Likewise, the review by Lacroix-Desmazes et al. [6] is not appropriate for citation as the investigators excluded data from studies on patients with SNR. 

In our review we noted that some patients desire shorter injection cycles, based on a survey which found that 45% of patients expressed preference for a treatment cycle of less than 10 weeks [11]. Although this view is frequently expressed by patients treated with BoNT, we certainly do not endorse or advocate for “booster” injections, which in the past have been associated with high frequency of immunoresistance [1]. Most studies examining the effects and safety of shorter inter-dose intervals used incobotulinumtoxinA [12,13,14], but more comparative data are needed before concluding that one product or another is associated with less immunogenicity or longer duration of action [15].

Many of the points raised in the commentary by Foster and Beard are echoed in our review [1]. Immunoresistance is a rare phenomenon and accounts for a small minority of patients found to be unresponsive to BoNT. The relationship between NAbs and SNR is unclear; however, it cannot be fully discounted. Comparison of NAb formation rates between formulations or between studies is challenging due to a variety of factors, including different patient populations and study methodologies, and varied assays used to measure clinical response and NAbs. We agree that clinical tests, such as the unilateral brow injection, while predictive of future unresponsiveness, do not necessarily reflect immunogenicity. Finally, we agree with the conclusion by the authors of the commentary that “BoNT therapy is a highly effective and important therapy for the treatment of a variety of neurological and non-neurological conditions.”
